# Antimicrobial and Antioxidant Properties of Chemically Analyzed Essential Oil of *Artemisia annua* L. (Asteraceae) Native to Mediterranean Area

**DOI:** 10.3390/life13030807

**Published:** 2023-03-16

**Authors:** Khalid Chebbac, Zineb Benziane Ouaritini, Abdelfattah El Moussaoui, Mohammed Chalkha, Soufyane Lafraxo, Yousef A. Bin Jardan, Hiba-Allah Nafidi, Mohammed Bourhia, Raja Guemmouh

**Affiliations:** 1 Laboratory of Biotechnology Conservation and Valorisation of Natural Resources, Faculty of Sciences Dhar El Mahraz, Sidi Mohamed Ben Abdellah University, Fez 30000, Morocco; 2 Laboratory of Natural Substances, Pharmacology, Environment, Modeling, Health and Quality of Life, Faculty of Sciences, Sidi Mohamed Ben Abdellah University, Fez 30000, Morocco; 3 Laboratory of Biotechnology, Environment, Agri-Food and Health, Faculty of Sciences Dhar El Mahraz, Sidi Mohamed Ben Abdellah University, Fez 30000, Morocco; 4 Engineering Laboratory of Organometallic, Molecular, Materials and Environment, Faculty of Sciences Dhar EL Mahraz, Sidi Mohamed Ben Abdellah University, Fez 30000, Morocco; 5 Department of Pharmaceutics, College of Pharmacy, King Saud University, Riyadh 11451, Saudi Arabia; 6 Department of Food Science, Faculty of Agricultural and Food Sciences, Laval University, Quebec City, QC G1V 0A6, Canada; 7 Laboratory of Chemistry and Biochemistry, Faculty of Medicine and Pharmacy, Laayoune 70000, Morocco

**Keywords:** *Artemisia annua* L. (Asteraceae), essential oil, chemical constituents, antimicrobial activity, antioxidant capacity

## Abstract

*Artemisia annua* (AA) is an aromatic plant belonging to the *Asteraceae* family, which has long been known for its several medicinal virtues. In addition, essential oils (EOs) extracted from AA have a wide range of therapeutic properties. Therefore, this study aimed to investigate the phytochemical composition, anti-microbial, and anti-oxidant properties of *Artemisia annua* essential oil (EOAA). EO was extracted, and its chemical constituents were ascertained by the use of GC-MS analysis. EOAA shows remarkable antioxidant capacities of DPPH free radical scavenging with an IC_50_ value of 29 ± 5.3 μg/mL and ferric reducing antioxidant power with an EC_50_ value of 9.21 ± 0.3 µg/mL, and it also has a good total antioxidant capacity of 911.59 ± 115.71 milligrams of ascorbic acid equivalence per gram of EO (mg AAE/g EO). Moreover, the in vitro antimicrobial screening results indicate that EOAA has shown promising antibacterial activity, especially against the *Escherichia coli* strain, and it also shows significant antifungal activity against *Fusarium oxysporum* and *Candida albicans* yeasts. Taken together, our findings highlight the importance of EOAA as a source of strong antioxidant and antimicrobial agents, which could be used as an alternative form to control free radicals and combat drug-resistant microbes.

## 1. Introduction

The emergence and spread of infectious diseases caused by bacteria and fungi have become a serious threat and an important cause of mortality of living beings [1,2]. The fight against pathogens is mainly performed through the use of antibiotics. However, the development of multidrug-resistant microorganisms has serious consequences on the healthcare system and makes the treatment of these infections a great challenge [3]. Furthermore, the imbalance between the generation and detoxification of reactive oxygen species leads to oxidative stress that poses serious risks to human health [4,5]. This phenomenon leads to oxidative damage of cell membranes, the degradation of several biological molecules, and causes many severe pathologies [6,7]. Consequently, it is crucial to find new powerful substances that can address the issues caused by free radicals and drug-resistant microorganisms.

To overcome these problems, scientists have paid more attention to natural products for the development of new drugs with high efficiency and low toxicity [8,9]. Medicinal and aromatic plants (MAP) are a gift of nature to mankind to help them lead a healthy and disease-free life [10]. The practice of harnessing natural products to cure and relieve pain and disease in both humans and domestic animals is as old as the history of human development [11]. Recently, there has been a growing interest in the utilization of natural medications as a means to reduce the risk of adverse effects and the expense of synthetic drugs [12]. 

The species of the family *Asteraceae* are among the natural plants that have been widely used for a long time in popular cooking all over the world owing to their excellent dietary value. It is especially consumed as an herbal tea by diabetics (Moroccan tea) and in salads, used as appetizers, spices, and condiments, as well as coloring agents in Moroccan and Chinese dishes [13]. They contain chemical constituents that serve as an important sources for the design and development of drugs competitive with synthetic products, and some of them are approved for clinical use [14].

The genus *Artemisia* comprises woody shrubs and herbs that grow mainly in semi-arid (steppe climate) regions. This genus includes more than 500 species as recorded in the literature [15]. It is distinguished by a great morphological and phytochemical variety and variability, which are related to the specimen’s various geographic origins [16,17]. Besides, many studies on the genus Artemisia are conducted to discover its evolutionary and taxonomic relationships [18,19]. Furthermore, the scientific importance of the *Artemisia* plant comes from its biologically active substances, such as sesquiterpenes and terpenoids (mainly monoterpenes in EOs) [20]. These phytochemical classes are recognized for a wide variety of interesting biological activities [21]. 

*Artemisia annua* (AA) is a herbal plant from the family Asteraceae (subfamily *Asteroideae*), native to the Old World (Eurasia, North Africa). *A*. *annua* has been widely used in Chinese folk medicine for many centuries [22]. This plant has been recognized for its many extraordinary medicinal properties. The extracts of *A*. *annua* demonstrate many interesting biological properties, such as having anti-malarial, anti-cancer, anti-inflammatory, and antiulcerogenic activities [23,24]. The medicinal efficacy of *A*. *annua* is attributable to the variety of active substances it contains, among which is the artemisinin, which is used to treat the chloroquine-resistant *Plasmodium falciparum* [25]. In addition, essential oils (EOs) extracted from AA have been extensively reported to have a wide range of therapeutic properties [26].

Relying on the aforementioned statement and ensuring the continuity of our ongoing research focused on the assessment of the biological and pharmacological properties of EOs of the genus *Artemisia* plant [9,27], we report in the current study the analysis of the chemical composition and the evaluation of the antimicrobial and antioxidant properties of the EO extracted from *Artemisia annua*, a native of Morocco.

## 2. Materials and Methods

### 2.1. Reagents and Chemicals 

All reagents and chemicals used, such as ammonium molybdate ((NH_4_)_6_Mo_7_O_24_), sodium phosphate, 2,2-diphenylpicrylhydrazyl radical (DPPH), quercetin, ascorbic acid, gallic acid, potassium ferricyanide (K_3_[Fe (CN)_6_]), ferric chloride (FeCl_3_), resazurin, agar, fluconazole, and trichloroacetic acid (TCA), were purchased from Sigma-Aldrich (St. Louis, MO, USA) and other commercial suppliers.

### 2.2. Plant Material Collection, Preparation, and EO Extraction

*Artemisia annua* was collected in October 2021 at the southern slopes of the Middle Atlas Mountains–Morocco (latitude: 33.76965638; longitude: 3.49256285; altitude: 741 m). The identification was conducted by a botanist Bari Amina under the voucher number AAN001JB241021 before being deposited in the university herbarium. After that, the plant’s aerial parts were sliced into medium-sized pieces and dried at room temperature in a chamber for two weeks. Next, the dried plant material was ground into a fine powder and subjected to hydro-distillation with a Clevenger-type apparatus for 2 h and 15 min until the total extraction of the oil. The resulting EO was desiccated over anhydrous sodium sulfate (Na_2_SO_4_) and preserved in amber worm bottles and stored at 4 °C until tested [28].

### 2.3. Analysis of the Chemical Composition by Use of GCMS

GC-MS analysis was used to identify the chemical composition of EOAA. Briefly, after diluting the EOAA in hexane (10:100 dilution), 1 µL was used for the identification and quantification of constituents by the use of gas chromatography–mass spectrometry (GC-MS-TQ8040 NX, brand Shimadzu, Tokyo, Japan), equipped with an apolar capillary column (RTxi-5 Sil MS-30m × 0.25 mm ID × 0.25 µm). The initial oven temperature program was maintained at 50 °C for two minutes and ramped at 5 °C/min until 160 °C for 2 min, then ramped at 5 °C/min up to 280 °C for 2 min. Notably, the analysis time was 50 min, and high-purity helium was employed as the carrier gas at a flow rate of 1 mL/min, with a split ratio of 1:20. The injection temperature was held at 250 °C, while that of the detector was held at 280 °C [29]. The injected volume of the EO sample was 1 μL, and the ion source temperature operated at 200 °C with an electron ionization energy of 70 eV utilizing a spectral range of m/z 40–650. Chemical compounds found in the EOAA were identified through the comparison of their mass spectra with those found in the MS library (using the NIST-MS Search Version 2.0 program). Additionally, their Kovats index was compared with the reference Adams database [30].

### 2.4. Antioxidant Activity 

#### 2.4.1. DPPH Test 

The DPPH assay was performed according to the method of Shams Moattar and co-authors, with slight modifications. [31] was used for the DPPH test. Briefly, from a methanolic solution of EOAA, a dilution series was prepared, and 100 µL of each of its concentrations was taken and placed in an Eppendorf tube. After that, 1000 μL of a methanolic solution of DPPH (0.005%) was added. Then, the mixture was stirred and incubated in the darkness for 40 min. Finally, the optical density was well measured at 517 nm using a Shimadzu 160-UV spectrophotometer virus a blank (negative control). Ascorbic acid, gallic acid, and quercetin were used as standards. DPPH free radical inhibition was determined as follows:

$$PI\;\left(\%\right)=\frac{\left(A0\right)-\left(A\right)}{\left(A0\right)}\times 100$$

**PI**: Percentage of inhibition, 

**A0**: Absorbance of the control, 

**A**: Absorbance of EOAA 

#### 2.4.2. Ferric Reducing Antioxidant Power (FRAP) Test

The reducing power of EOAA was ascertained following our previously described procedure with slight modifications [27]. Briefly, 0.05 mL of EOAA were added to 0.2 mL of phosphate buffer (200mM, pH = 6.60) and 0.2 mL of (NH_4_)_6_Mo_7_O_24_ (1% each). After shaking, the solution was left in a water bath at 50 °C for 25 min. Next, 0.6 mL of water stirred with 0.12 mL of 0.1% FeCl_3_ and 0.2 mL of trichloroacetic acid (10% TCA). The absorbance of the mixture was then determined with a UV-visible spectrophotometer at 700 nm. Efficacy concentration (EC-50) was used to report results. 

#### 2.4.3. Total Antioxidant Capacity (TAC) Test 

TAC of EOAA was conducted as follows; one milliliter of reagent prepared by use of sodium phosphate, sulfuric acid, and (NH_4_)_6_Mo_7_O_24_ was mixed with 0.025 mL of EOAA [32]. Next, the tubes were stirred, screwed, and incubated at 96 °C for 1 h and 25 min, and then they were allowed to cool at room temperature. The optical density of the mixture and positive controls was measured at 695 nm by means of a UV-visible spectrophotometer and compared to a blank consisting of 25 µL of methanol instead of EOAA. Butylhydroxytoluene (BHT) and quercetin were used as standards, and the TAC was expressed as milligrams of ascorbic acid equivalence per gram of EO (mg AAE/g EO).

### 2.5. Antimicrobial Activity 

#### 2.5.1. Microbial Strains Tested 

The antimicrobial activity of EOAA was performed against three fungal strains, which are *Candida albicans* (ATCC 10231), *Aspergillus niger* (MTCC 282), and *Fusarium oxysporum* (LBEAH/FS/17), as well as three Gram-negative bacterial strains, which are *Escherichia coli* (K12), *Klebsiella pneumoniae*, *Salmonella* sp., and three Gram-positive bacterial strains, which are *Staphylococcus aureus* (ATCC 6633), *Bacillus subtilis* (DSM 6333), and *Bacillus cereus*.

#### 2.5.2. Agar Diffusion Method

The Agar diffusion method was used to assess the antimicrobial activity of EOAA as reported previously [33]. Briefly, bacterial and fungi suspensions were prepared to be 0.5 McFarland approximately 1–2 × 10^5^ CFU/mL. Next, bacteria and fungi were seeded in Petri dishes inoculated with Mueller-Hinton Agar (MHB) and yeast-peptone-glucose (YPG) extract media, respectively. After that, 6 mm Wattman N°4 paper discs were placed on the agar surface of Petri dishes and soaked with 10 µL of EOAA and kanamycin (233398) used as a positive control (30 µg). Then, the plates were placed in the dark and incubated for 24 h at 37 °C for bacterial strains and 48 h at 30 °C for fungal strains, especially, and *Fusarium oxysporum* and *Aspergillus niger* were incubated for seven days [34]. The zones of inhibition were measured and expressed in millimeters (mm).

#### 2.5.3. Minimal Inhibitory Concentration (MIC)

To determine the MIC of EOAA against the aforementioned microbial strains, the microdilution method (96-well microplate) was used as described by Sadiki [35]. The emulsifier used (0.15% (*w*/*v*) bacterial agar) was mixed with Mueller-Hinton broth (MHB). EOAA was serially diluted in agar-supplemented broth at concentrations ranging from 870 to 1.4 µg/mL. Then, 50 μL of bacteria (10^6^ CFU/mL) was applied. Finally, bacterial growth was visualized by turning resazurin from purple to pink.

Similarly for *Candida albicans*, the MIC values were determined according to the protocol described by Clinical and Laboratory Standards Institute (CLSI) [36]. The EOAA dilution was prepared in YPG broth containing 0.15% (*w*/*v*) agar. Then, the fungal inoculum (50 µL containing 10^3^ CFU/mL) was added to each well of the plate, which was incubated at 30 °C for 48 h.

For the *Fusarium oxysporum* and *Aspergillus niger* strains, a similar protocol was followed, using 0.15% sauton agar as the culture medium and incubating for 72 h. The culture medium was 0.15% sauton agar. The experiments were repeated multiple times, and results are expressed as means [27]. 

### 2.6. Statistical Analysis 

The data are displayed as the mean value and the standard deviation value (mean ± SD). In order to carry out the analysis, GraphPad Prism 9 was utilized. In order to make comparisons, we used analysis of variance (ANOVA), and then we used Tukey’s highly significant difference test. A statistically significant difference was thought to exist when the *p*-value was less than 0.05.

## 3. Results 

### 3.1. Yield and Chemical Composition

**Figure 1:**
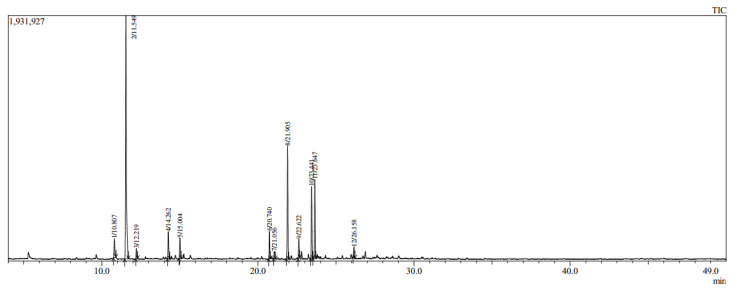
Chromatographic profile of EOAA profiled by GC-MS analysis.

The yield of Eos recovered from the aerial parts of *A*. *annua* was about 0.51%. This rate is comparable to that found in the genus Artemisia e.g., *A*. *mesatlantica,* and *A*. *annua*, which grow in the Mediterranean area, and they have an essential oil yield of about 0.5% [37,38]. Similarly, *A*. *aragonensis*, *A*. *negrei*, and *A*. *campestris* have around 1.2% of the EOs [9,27,39]. In addition, twelve compounds were identified by GC-MS analysis with a predominance of artemisia ketone (43.19%) followed by a Caryophyllene (15.75%) and β-Selinene (10.32%). The gas chromatogram of EOAA is provided in Figure 1, and the detailed qualitative and quantitative chemical composition of EOAA is presented in Table 1.

### 3.2. Antioxidant Activity of EOAA

**Figure 2:**
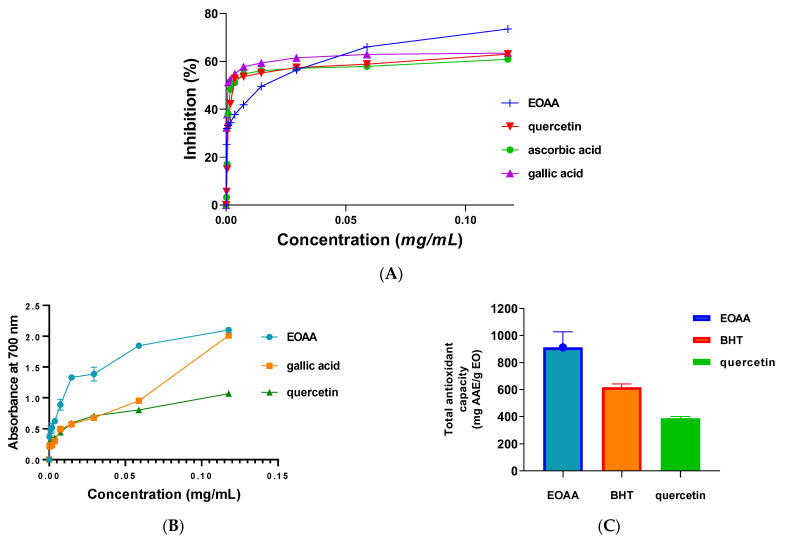
Antioxidant capacity using DPPH (**A**), FRAP (**B**), and TAC assays (**C**).

The effectiveness of the free radical scavenging capability of EOAA was tested with the DPPH assay. The results showed that EOAA possessed potent antioxidant power with an IC_50_ value of 29 ± 5.3 μg/mL, lower than of the positive controls, quercetin (IC_50_ = 43 ± 0.5 μg/mL), and ascorbic acid (IC_50_ = 42 ± 0.9 μg/mL). However, this value is higher than that of the antioxidant gallic acid (IC_50_ = 2.2 ± 0.6 μg/mL) (Figure 2A). Notably, a smaller value of IC_50_ indicates potent antioxidant activity of the tested agent [27]. Furthermore, according to the ferric reducing power assay (FRAP), EOAA exhibited an EC_50_ value in the range of 9.218 ± 0.3 µg/mL, while gallic acid and quercetin recorded EC50 values of 17.28 ± 0.4 µg/mL and 20.87 ± 1.1 µg/mL, respectively (Figure 2B). Besides, the total antioxidant capacity of EOAA was 911.59 ± 115.71 mg AAE/g EO, whereas quercetin and BHT used as antioxidant standards recorded 388.744 ± 12.289 and 616.244 ± 25 mg AAE/g EO, respectively (Figure 2C).

### 3.3. Antimicrobial Activity of EOAA

**Figure 3:**
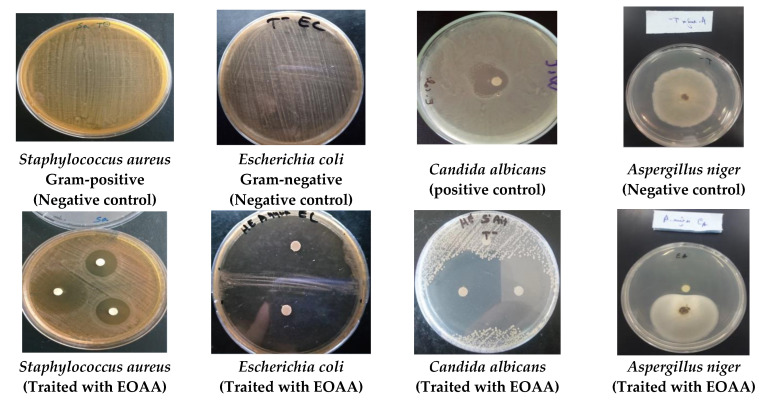
Photo displaying the effects of EOAA on the tested bacteria and fungi (the number of discs in the Petri dishes signifies the number of repetitions).

The results obtained from the antimicrobial screening of EOAA and standard drugs against Gram-negative bacteria, Gram-positive bacteria, and the fungi *Fusarium oxysporum*, *Aspergillus niger,* and *Candida albicans* are provided in Table 2, Table 3 and Table 4 and Figure 3. The tested microbial strains are characterized by their resistance and high pathogenicity [27,40]. The outcomes revealed strong antimicrobial capacity of EOAA against Gram-negative bacteria, especially *Escherichia coli* with an inhibition zone of 75.67 ± 2.05 mm and MIC of 5.34 ± 0.50 µg/mL (Table 2 and Table 3). Likewise, EOAA showed a MIC value of 1.09 ± 00 µg/mL and an inhibition zone of 16 ± 1 mm against the *Salmonella* sp. strain. However, *Bacillus cereus* as Gram-positive bacteria was less sensitive to EOAA, which had an inhibition zone of 11 ± 1 mm and a MIC value of 5.44 µg/mL (Table 2 and Table 3 and Figure 3).

The EOAA showed also significant activity against the fungal strains as compared to the antifungal drug. According to the findings, EOAA was effectively inhibited by the fungi *Fusarium oxysporum*, *Aspergillus niger,* and *Candida albicans*, with inhibition zones of 57.67 ± 2.08, 45.71 ± 4.06, and 45.67 ± 2.05 mm, respectively (Table 4). Similarly, the EOAA showed MICs values of 14.38 ± 2.37 µg/mL and 21.75 ± 0.34 µg/mL against *Fusarium oxysporum* and *Aspergillus niger*, respectively, while the MIC value obtained against *Candida albicans* was 3.12 ± 00 µg/mL (Table 4 and Figure 3). All bacterial strains tested showed resistance to the antibiotics used as positive controls. However, *Fusarium oxysporum*, *Aspergillus niger,* and *Candida albicans* showed sensitivity to fluconazole, with an inhibition zone of 23.33 ± 1.25, 36.12 ± 1.70 and 34.67 ± 2.62 mm and a MICs values of 2.78 ± 0.12, 2.01 ± 0.01 and 3.47 ± 0.05 µg/mL, respectively (Table 4 and Figure 3). 

## 4. Discussion

The demand for essential oils derived from natural plants has increased over the years owing to their use as medicines against many severe diseases and their low toxicity [41]. For this purpose, we examined the antimicrobial and antioxidant properties of EOs from *Artemisia annua* L. indigenous to the Mediterranean area. The extraction of the EO that we carried out allowed us to obtain a yield of approximately 0.51%. We have noticed that the yield changes as a function of the use of the leaves or flowers, the site of culture, the origin of the seeds, and the moment of the harvest. Importantly, the variation of chemical compounds is generally influenced by the drying conditions, harvest season, geographical location, fertilization, soil pH, as well as the chemotype, the part of the plant used, genotype, or extraction method [42]. The analysis of EOAA showed a high content of oxygenated monoterpenes (55.35%) and hydrocarbon sesquiterpenes (13.43%). However, oxygenated monoterpenes (1.62%) were detected in lower quantity while recording the absence of oxygenated monoterpenes. Our results agree with a previous study by Verdian, which showed that oxygenated monoterpenes (83.7%) and sesquiterpenes (12.5%) constitute the major compounds in Iranian *Artemisia annua* EOs [43]. Additionally, we have observed that EOAA are rich in active substances, such as artemisia ketone (43.19%), caryophyllene (15.75%), β-selinene (10.32%), and germacrene D (9.56%) according to GC-MS analysis. Most of these components are present in the EOs extracted from the genus *Artemisia* collected in the Mediterranean area (*A*. *arborescens*, *A*. *caerulescens* subsp. and *A*. *annua*) [44,45], as well as in other countries, such as India, France, Turkey, and North America [46,47,48,49]. In addition, compounds in essential oils from *A*. *mesatlantica* endemic to Morocco are slightly similar to those revealed in EOAA [38,50]. Importantly, many compounds in EOAA are also detected in nearby plant species, including *Artemisia herba-alba* L., *Artemisia pontica* L., and *Artemisia absinthium* L, which is in agreement with our study. Notably, 1,8 cineol, camphor, and borneol are among the main compounds of EOAA, and they have been widely found in the EO of the genus Artemisia [51]. There are few reports on the analysis of EOs from Mediterranean plants in the literature [44]. Interestingly, due to the presence of different chemotypes within species, variations in essential oil concentration between subspecies have been reported. Notably, GC-MS showed some special compounds in Moroccan *A*. *annua*, which may confirm its indigenous to this country [52]. Thus, it seems that EO possesses a chemical polymorphism, with different chemotypes related to intrinsic and extrinsic factors of the species [53].

The presented result here agrees with those reported by Chirane et al., who showed that artemisia ketone detected in EOAA had a good antioxidant power when compared to BHT. Because of this fitting, the antioxidant potency of EOAA may be due to its richness in artemisia ketone [54]. Artemisia ketone showed higher activity than 1,8-cineole and camphor in the DPPH test, as well as in the recorded literature [45,55]. Previous studies showed that the antioxidant effect of EOs is frequently related to their major compounds [56,57]. The synergistic effect of minor chemicals in EOs can determine their antioxidant powder of EO [27,58]. In general, OEs rich in oxygenated monoterpenes have a more pronounced antiradical activity than those with hydrocarbon terpenes [52]. Importantly, the antioxidant results are in agreement with those found in previous works, where it was reported that the genus Artemisia has promising antioxidant activity by all bioassays used, such as DPPH, *β*-carotene bleaching, and total antioxidant capacity [9].

Concerning the antibacterial power, our results are comparable with those reported elsewhere [59,60], which showed that EOs from the genus Artemisia possess antimicrobial potential. EOAA showed better antibacterial activity when compared to Iranian EOAA [61]. The antimicrobial action demonstrated by EOAA is mostly related to its high content of oxygenated monoterpene and hydrocarbon sesquiterpenes (55.33 and 43.03% respectively), which are known for their antibacterial action [61,62]. The current results agreed with those reported by Habibi et al. [63], who showed that EOAA had significant antibacterial capacity against bacterial strains, particularly versus *Escherichia coli*, *Staphylococcus aureus,* and *Bacillus subtilis*. In addition, our findings are in accordance with the findings of a number of previous research efforts, such as the one conducted by Radulovic et al. [55], who reported that the compound artemisia ketone possessed antibacterial power vs. pathogenic microorganisms. Some studies indicated that Gram-positive bacteria are more sensitive to the effect of Eos, unlike Gram-positive bacteria [29]. The findings of the current research, on the other hand, suggested that the difference in the cell walls of Gram-positive and Gram-negative bacteria was not a significant factor in determining the antibacterial activity of EOAA, since *Escherichia coli* was the most sensitive to essential oil with a zone of inhibition of 75.67 mm and minimum inhibitory concentration of 5.34 μg/mL. These results may be explained by the broad-spectrum activity of EOAA. According to the previous findings, the essential oils of the genus Artemisia is effective against Gram-negative (*Escherichia coli* 57, *Escherichia coli* 97, *Klebsiella pneumonia*, and *Pseudomonas aeruginosa*) and Gram-positive (*Staphylococcus aureus*) bacteria, with maximum inhibition zones ranging from 18–37 mm and MIC values ranging from 3.25 to 12.5 mg/mL, respectively [9].

The antibacterial properties of EOs can be attributed to their lipophilic nature, which allows them to effectively infiltrate into bacteria cells. In this regard, it was found that hydrocarbons in EOs (43.03% of EOAA) are predominantly accumulated in cytoplasmic membranes, which leads to impaired membrane permeability and, ultimately, the rapid death of microorganisms [64]. The phytochemicals in EOAA (artemisia ketone, caryophyllene, β-selinene and germacrene D) may act synergistically, rather than individually, as previous research showed that the antibacterial activity of EOs is more potent than their compounds examined individually [65,66].

The antifungal activity of EOAA can be explained by the synergistic effect between the different chemical substances of essential oil, notably artemisia ketone, caryophyllene, and β-selinene, which are often responsible for the antifungal activity, as well as the minority compounds that can also contribute significantly to this activity [67]. Some studies reported that the antimicrobial activity of EOs may be more important than that of their major compounds when tested separately [58]. Importantly, the activity of the essences is frequently resultant from synergic effect of minor components in EOs. Moreover, a proportional correlation between the presence of oxygenated terpenes and the antimicrobial potency is reported in the work of Agour [68], which is in agreement with our results regarding oxygenated monoterpenes in EOAA, found to be 55.35%. 

## 5. Conclusions

The EO extracted from *Artemisia annua* grown in the Middle Atlas region of Morocco showed potent antioxidant and antimicrobial activities against antibiotic resistant microbes. The remarkable bioactivities observed can be attributed to the chemicals found in the EO, especially the main constituents, such as artemisia ketones (43.19%), caryophyllene (15.75%), β-selinene (10.32%), and germacrene D (9.56%). Altogether, the explored EOAA can be considered as a possible alternative to combat drug-resistant microbes and oxidative stress. Additionally, it will be crucial to conduct in vitro and in vivo studies on other biological targets, as well as to further assess any possible adverse effects on non-target organisms.

## Figures and Tables

**Table 1:** Chemical constituents identified in EOAA by GC/MS analysis.

P	R.T.	Name	Area%	R. I.		M.W. (g/mol)	Chemical Structure	Chemical Classes
				Lit	Obs			
1	10.807	1,8-cineole	3.21	1031	1059	154	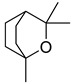	M.O
2	11.549	artemisia ketone	43.19	1048	1042	152	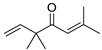	M.O
3	12.219	artemisia alcohol	1.48	1071	1068	154	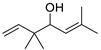	M.O
4	14.262	camphor	4.41	1127	1121	152	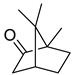	M.O
5	15.004	borneol	3.07	1152	1138	154.25	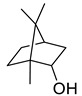	M.O
6	20.740	copaene	3.52	1363	1221	204	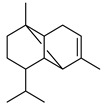	S.H
7	21.056	*γ*-cadinene	1.25	1490	1435	204	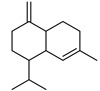	S.H
8	21.905	caryophyllene	15.75	1440	1494	204	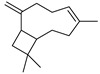	S.H
9	22.622	*β*-farnesene	2.62	1442	1440	204	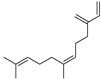	S.H
10	23.441	germacrene D	9.56	1491	1515	204	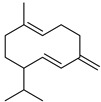	S.H
11	23.647	*β*-selinene	10.32	1473	1469	204	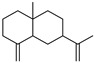	S.H
12	26.158	caryophyllene oxide	1.62	1549	1507	20	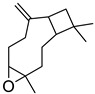	S.O
Chemical classes								
Oxygenated Monoterpenes (M.O)								55.35%
Oxygenated Sesquiterpenes (S.O)								1.62%
Sesquiterpene Hydrocarbons (S.H)								43.03%
Total Identification								100%

P: Peak; R.T: Retention time; Obs: Observed; Lit: Literature; R.I: Retention index. M.W.: Molecular weight.

**Table 2:** Antibacterial activity of EOAA and Kanamycine by use of inhibition zone diameter (mm).

Compound	Gram-Positive Bacteria			Gram-Negative Bacteria		
	*Bacillus* *subtilis*	*Bacillus cereus*	*Staphylococcus aureus*	*Escherichia coli*	*Klebsiella pneumoniae*	*Salmonella* sp.
EOAA	37.0 ± 0.0 ^a^	11.0 ± 1.0 ^d^	23.33 ± 5.86 ^c^	75.67 ± 2.05 ^e^	22.33 ± 1.15 ^c^	16.0 ± 1.0 ^d^
Kan	Rst ^b^	Rst ^b^	Rst ^b^	Rst ^b^	Rst ^b^	Rst ^b^

Rst: Resistant (inhibition zone diameter ≤ 15 mm); Kan: Kanamycine; EOAA: *A*. *annua* essential oil. Values that have the same sign (^a^, ^b^, ^c^, …) do not represent a significant difference.

**Table 3:** Antibacterial activity of EOAA and Kanamycin by use of minimum inhibitory concentration (µg/mL).

Compound	Gram-Positive Bacteria			Gram-Negative Bacteria		
	*B*. *subtilis*	*B*. *cereus*	*Staphylococcus aureus*	*Escherichia coli*	*K*. *pneumonia*	*Salmonella* sp.
EOAA	5.64 ± 0.97 ^a^	5.44 ± 0.0 ^a^	4.98 ± 1.82 ^b^	5.34 ± 0.5 ^a^	5.44 ± 0.0 ^a^	1.09 ± 0.0 ^d^
Kan	2.65 ± 0.45 ^c^	2.75 ± 0.01 ^c^	2.44 ± 0.64 ^c^	1.78 ± 0.35 ^d^	2.33 ± 0.27 ^c^	1.04 ± 0.0 ^d^

Kan: Kanamycine; EOAA: *A*. *annua* essential oil. Values that have the same sign (^a^, ^b^, ^c^, …) do not represent a significant difference.

**Table 4:** Antifungal activity of EOAA and fluconazole by use of minimum inhibitory concentration (MIC) and inhibition zone diameter.

	MIC (μg/mL)		Inhibition Diameter (mm)	
Fungal Strains	EOAA	Flu	EOAA	Flu
*Candida albicans*	3.12 ± 0.00 ^a^	3.47 ± 0.05 ^a^	45.67 ± 2.05 ^c^	34.67 ± 2.62 ^e^
*Fusarium oxysporum*	14.38 ± 2.37 ^b^	2.78 ± 0.12 ^a^	57.67 ± 2.08 ^d^	23.33 ± 1.25 ^f^
*Aspergillus niger*	21.75 ± 0.34 ^f^	2.01 ± 0.01 ^a^	45.71 ± 4.06 ^c^	36.12 ± 1.70 ^e^

Flu: Fluconazole; EOAA: *A*. *annua* essential oil. Values with the same sign (^a^, ^b^, ^c^, …) do not represent a significant difference.

## Data Availability

Not applicable.
